# Oral health care for frail older adults in nursing homes from a management perspective: a survey-based study

**DOI:** 10.1186/s12903-025-07327-x

**Published:** 2025-12-14

**Authors:** Lisa Bellander, Helle Wijk, Pia Andersson, Catharina Hägglin

**Affiliations:** 1 Department of Behavioral and Community Dentistry, Institute of Odontology, Sahlgrenska Academy, University of Gothenburg, Gothenburg, 405 30 Sweden; 2 Centre of Gerodontology, Public Dental Service, Region Västra Götaland, Gothenburg, 402 33 Sweden; 3 Institute of Health and Care Sciences, Sahlgrenska Academy, University of Gothenburg, Gothenburg, 405 30 Sweden; 4 Department of Architecture and Civil Engineering, Chalmers University of Technology, Gothenburg, 412 96 Sweden; 5 Department of Quality Strategies, Sahlgrenska University Hospital, Gothenburg, Region Västra Götaland 413 45 Sweden; 6 Department of Oral Health, Faculty of Health Sciences, Kristianstad University, Kristianstad, 291 88 Sweden

**Keywords:** Geriatric nursing, Management professionals, Oral health, Oral hygiene, Quality register, Risk assessments, ROAG, Senior alert

## Abstract

**Background:**

Oral diseases and poor oral hygiene are prevalent among frail older adults in nursing homes. Despite well-known organizational barriers such as low prioritization, time and staff shortages, and lack of routines and training, research on the perspective of nursing home management remains limited in this area.

**Purpose:**

To investigate the views and experiences of professionals in leadership roles regarding oral health needs and routines as well as barriers and facilitators in providing effective oral care for frail older adults in nursing homes.

**Methods:**

A web-based survey was sent to 1,526 nursing home professionals (managers, coordinators and registered nurses) in Sweden. The survey comprised a 52-item questionnaire covering oral health needs, oral care barriers and facilitators, routines, education, collaboration with dental care services, and the use of the quality register Senior Alert, designed to support a preventive approach in nursing care and the Revised Oral Assessment Guide-Jönköping (ROAG-J).

**Results:**

Responses were received from 166 managers, 55 coordinators and 243 nurses, yielding a 32% response rate. About half of the respondents perceived residents’ oral health as poor, and 83% reported that most residents required assistance with oral care. The main barriers were difficulties for nursing staff to perform oral care and residents’ unwillingness to participate, often due to dementia. Increased and regular training in oral care for nursing staff was viewed as the most important facilitator; however, only 41% of respondents reported that their staff had received training regularly. The Senior Alert register and the ROAG-J were widely used and generally perceived as effective. Oral health was also frequently discussed during regular Senior Alert team meetings, with a high level of management involvement.

**Conclusions:**

The significant and complex oral care needs of older adults in nursing homes, coupled with the fact that nursing staff often lack adequate training, stress the importance of management allocating resources, ensuring regular staff training and strengthening collaboration with dental care services. Senior Alert’s structured approach seems to have the potential to enhance the engagement of nursing home professionals in managing residents’ oral health.

**Supplementary Information:**

The online version contains supplementary material available at 10.1186/s12903-025-07327-x.

## Introduction

Oral diseases, including dental caries and periodontitis, are highly prevalent worldwide [1].

As a result of improved dental care and better health-related behaviours, such as the use of fluoride toothpaste, many adults maintain good oral health and retain their natural teeth well into old age [2, 3]. Paradoxically, this positive trend of more individuals being dentate has also led to an increased risk of developing caries and periodontitis, particularly among older adults [3, 4]. Risk factors contributing to oral diseases in older ages include reduced saliva flow, difficulties with oral self-care due to functional or cognitive impairments, complex dental restorations and lower dental care utilization [5–8]. Oral health problems can lead to pain, malnutrition, lower life satisfaction and social isolation [9, 10]. Poor oral health can also contribute to the development of frailty [11], a progressive condition common in advanced age and associated with reduced physical function, greater care needs, and higher mortality risk [12]. Frailty may, in turn, lead to a deterioration of oral health [10]. Furthermore, there is a well-established association between oral health and systemic diseases such as coronary heart disease, diabetes and respiratory conditions [13–15].

In nursing homes, where most residents are frail, have multimorbidity (the coexistence of two or more chronic conditions) and/or cognitive impairments, poor oral health is very common [16, 17]. Residents’ oral health care needs are more often unmet and neglected in comparison to other care needs [18, 19]. Caregivers have particularly pointed out difficulties in providing oral care, especially to individuals with dementia, who often refuse assistance [18–20]. Additionally, nursing staff have been shown to lack knowledge and skills in oral care [18, 19, 21]. Other barriers at the organizational level include the absence of oral health routines and policies as well as challenges in accessing and collaborating with dental care services [22, 23]. Overall, there is therefore an urgent need to establish systematic, multidisciplinary preventive strategies to maintain good oral health for older adults in nursing homes [3].

In Swedish nursing homes, the national quality register Senior Alert has been widely used since 2008 to prevent common age-related risks, including oral health issues as well as falls, malnutrition, pressure ulcers, and bladder dysfunction [24]. In Senior Alert assessments are made by registered nurses and nurse assistants using established assessment instruments. To help nursing staff detect oral health problems, the validated screening instrument Revised Oral Assessment Guide (ROAG) is used upon admission to nursing home and is recommended to be performed at least every six months [25, 26]. Within Senior Alert, ROAG has been slightly modified and renamed ROAG-J, and this version also includes suggestions for preventive actions. The ROAG-J contains nine assessment items (voice, lips, mucous membranes, tongue, gums, teeth, dentures, saliva and swallowing) and is graded as follows: 0 for not applicable, 1 for healthy conditions, 2 for moderate risk and 3 for severe risk of oral health problems (see Additional file 1). When risks are detected with the ROAG-J, underlying causes, preventive actions and referral and contact with dental care services as well as regular follow-ups are planned and recorded in the Senior Alert system. The screening takes only 3–4 min to perform. To ensure the reliability and consistency of ROAG-J assessments, personnel should ideally receive appropriate training in its use [17, 27]. The level and type of training may vary across nursing homes. In 2023, approximately 70,000 nursing home residents were assessed for risks in Senior Alert [28], which accounts for about 85% of all nursing home residents that year [29]. Of these, around 90% also received a ROAG-J assessment [28].

In our previous qualitative study, nursing staff perceived that the inclusion of oral health in Senior Alert and the use of the ROAG-J highlighted the importance of oral health and helped them work more systematically [21]. However, they also felt that managers did not prioritize oral health, which resulted in unclear routines and responsibilities as well as insufficient training to perform the oral assessment and provide oral care [21]. Research exploring managerial perspectives on the prevention of poor oral health among older adults in nursing home settings also remains scarce.

The aim of the present study was therefore to explore the views and experiences of professionals in leadership roles (managers, coordinators, and registered nurses) in nursing regarding:

- oral health care needs, barriers, and facilitators in daily practice;
- oral health routines, oral care training, and collaboration with dental care services;
- the quality register Senior Alert and the oral assessment instrument ROAG-J.

The study also aimed to examine how these perceptions varied by professional roles, type of nursing home, oral health education, and use of Senior Alert and the ROAG-J.

## Materials and methods

### Study design

This descriptive cross-sectional study was conducted using an anonymous web-based questionnaire.

### Study population

The sample targeted professionals in leadership roles in nursing care (managers, coordinators and registered nurses) working at nursing homes or dementia care facilities for older adults who require a high level of care and assistance in daily activities. Managers, coordinators and registered nurses working in short-term care, home care or home healthcare were excluded. Invitations to participate were sent to all 55 municipalities across two regions in western Sweden: a larger region with 49 municipalities (Västra Götaland) and a smaller one with six municipalities (Halland). Approximately 360 nursing homes, both publicly and privately owned, were eligible for inclusion.

In Sweden, registered nurses hold a leadership role in nursing homes and are responsible for overseeing healthcare in nursing homes, while nurse assistants or care aides provide support and assistance with daily care, including personal hygiene and oral care. Both registered nurses and nurse assistants conduct risk assessments in Senior Alert, including the ROAG-J [21]. In nursing homes, certain nurse assistants can sometimes serve as oral health representatives. To do so, they should have enhanced competence and an interest in oral health to support untrained or new staff.

Most care unit managers in nursing homes in Sweden hold a university degree and oversee approximately 50 employees, of which the majority are nurse assistants and nurse aides with or without formal education. The managers are responsible for organizational tasks including managing staff, providing ongoing education and overseeing the development of care routines [30]. The final leadership role is that of coordinators or team leaders. These are often experienced nurse assistants with delegated organizational responsibilities, such as assigning daily tasks, supervising and supporting nursing staff, and acting as a link between the team, relatives and managers.

### Setting

In Sweden, approximately 10% of people aged 80 and older reside in nursing homes, and around 70% of these have a dementia diagnosis [31, 32]. The majority (about 80%) of nursing homes are publicly owned and operated by municipalities [31]. Data from the Senior Alert public database indicate that approximately 80% of the nursing homes eligible for inclusion in this study were using the quality register in 2023 [28]. All nursing home residents receive support from social services provided by municipalities, while most also receive healthcare from the regional authorities. These two sectors operate under different legal frameworks and use separate medical record systems. Registered nurses work within the legal frameworks of healthcare, while managers, coordinators and nurse assistants operate under social services regulations.

The availability of dental care services (such as dentists and dental hygienists) in nursing homes varies by municipality, with both private and public dental providers offering domiciliary dental care. Frail older adults with certain illnesses, disabilities or a major need for nursing care can receive dental care subsidies provided by the regions. This support includes dental care at a very subsidized price as well as an annual free oral health assessment (not ROAG-J) and oral hygiene advice in their home provided by a dental hygienist [33].

### Questionnaire

Since no reliable and valid instrument was available to address the study aims, the authors developed a questionnaire based on previous research, professional experience, and the survey methodology of Wenemark [34], who also reviewed and provided guidance on the questionnaire items. The self-administered online questionnaire included 52 items and was designed using the web-based software Webropol 3.0 Survey & Reporting [35]. A detailed description of the survey questionnaire is provided in an additional file (see Additional file 2).

The questionnaire was divided into five sections: (A) demographics (13 items); (B) oral health care needs, barriers and facilitators to oral care (4 items); (C) oral health routines, oral care training and cooperation with dental care services (14 items); (D) Senior Alert (11 items); and (E) the ROAG-J (10 items). The questionnaire featured various types of items and response options. Since no single validated instrument matched the study’s specific aims, items were inspired by multiple sources or fully developed by the authors, and different Likert scales were applied, tailored to the content and purpose of each question. To assess perceived barriers to oral care in nursing homes, respondents were presented a list of predefined barriers based on previous research and asked to rate the extent to which they experienced each barrier on a 5-point Likert scale, ranging from ‘Not at all’ to ‘Completely’, with the additional option ‘No opinion’. The questionnaire also utilized a 5-point Likert scale (ranging from ‘Always’ to ‘Never’) for five items in section C, and a 4-point Likert scale (ranging from ‘Very good/well/easy’ to ‘Very poor/poorly/difficult’) for six items, all of which included an additional option for ‘Don’t know’ or ‘No opinion’.

Five items were open-ended questions. All items, except for the open-ended questions, were mandatory to complete in the survey. However, some filter questions were used, as certain items were only relevant for participants who, for example, actively worked with Senior Alert or had experience of performing the ROAG-J. In this study, “nursing staff” as referred to in some questionnaire items means nurse assistants or care aides who work most closely with the residents.

The questionnaire was pilot tested with two managers and four registered nurses, who were asked to evaluate whether the items were understandable and relevant as well as to suggest any changes. The review resulted in minor modifications in the phrasing of some items and adjustments to the layout. One of the participants in the pilot test met the inclusion criteria and was therefore part of the sample.

### Data collection

All local authority senior medical advisers in the two regions (Västra Götaland and Halland) were contacted via email, informed about the study and asked to notify relevant nursing home personnel within their municipality about the study and the importance of completing the survey. Contact information (email addresses) for managers, coordinators and registered nurses working in nursing homes, including dementia facilities, was requested and obtained from municipal administrative personnel, unit managers for home healthcare, the local authority senior medical advisers or directly from nursing home managers.

Of the 55 municipalities, two declined to participate due to reorganization, and two others could not be reached. All four were smaller municipalities, each comprising 1–7 nursing homes. In addition, some of the contact information was incomplete in four of the municipalities included in the study, mainly in the form of missing email addresses for nurses.

The questionnaire was distributed to the potential participants via email, including information about the study and an invitation to complete the questionnaire through a link. Participation was anonymous, and no information identifying individual nursing homes was collected. In total, the web survey was sent to 1,526 individuals: 524 managers, 183 coordinators and 819 registered nurses. The online survey was open for one month, from September to October 2024. Three reminders were sent to non-respondents at one-week intervals.

### Data analysis

Descriptive statistics were presented as frequencies and percentages. Non-parametric methods were used to assess group differences. The Mann–Whitney U test was applied for comparisons between independent groups on ordinal variables and Fisher’s exact test (two-sided) for categorical variables. For multiple-group comparisons, such as professional roles, the Kruskal–Wallis or Pearson Chi-Square tests were employed. When significant group differences were detected with the Kruskal–Wallis or Pearson Chi-Square test (< 0.05), pairwise comparisons were subsequently conducted using the Mann–Whitney U test or Fisher’s exact test. The response options ‘Don’t know’ and ‘No opinion’ were excluded from all inferential statistical analyses to ensure meaningful group comparisons. However, differences between professional groups regarding the proportion of ‘Don’t know’ responses, which in some cases were considerable, are presented descriptively in the results section. The frequency of oral care training for nursing staff was dichotomized into *regular* (‘every year’ and ‘every other year’) and *irregular* (‘less frequently than every other year’ and ‘never’). For all other variables, ordinal response categories were retained in the analyses but presented as dichotomized in tables and figures.

Group comparisons were conducted based on the following background variables: professional role, oral health education (received during professional education and/or at the workplace), type of nursing home (dementia care facilities/wards vs. general nursing homes), use versus non-use of the quality register Senior Alert and the oral assessment ROAG-J, and frequency of oral care training for nursing staff (regular vs. irregular).

For the open-ended items in the questionnaire, a summative content analysis was conducted [36]. The responses were reviewed, keywords were identified and categorized, and the frequency and percentages of responses were calculated and described narratively.

All statistical tests were two-tailed, and a *p*-value < 0.05 was considered statistically significant. Analyses were conducted using IBM SPSS Statistics (IBM Corp., Armonk, NY, USA), version 28.

## Results

### Final sample and response rate

The questionnaire was sent to 1,526 individuals, of whom 526 responded. Of these, 62 respondents (12%) were excluded for not meeting the inclusion criteria: 61 did not work in nursing homes and one did not have the requested professional role. After these exclusions, the final response rate was 32%.

The final sample consisted of 464 respondents. In total, 86% worked in the larger region, Västra Götaland, and 79% were employed in publicly owned nursing homes. Response rates were comparable across the professional groups (managers 32%, coordinators 30% and nurses 30%), and also similar across the two regions (Halland 71% vs. Västra Götaland 69%), but noticeably higher for publicly owned nursing homes compared to privately owned ones (73% vs. 46%).

### Characteristics of the respondents

In their professional education, 45% of managers had healthcare education, with approximately 65% being registered nurses or nurse assistants. Among the managers without a healthcare education, 40% were social workers. Of the coordinators, around 80% were nurse assistants. Table 1 provides descriptive characteristics of the respondents, including age, gender and years of experience in the profession, presented both by professional role and in total.

**Table 1:** Respondents’ characteristics according to professional role and in total

	Managers (*n* = 166)	Coordinators (*n* = 55)	Nurses (*n* = 243)	Total (*n* = 464)
Gender, *n* (%)				
Female	155 (93.5)	52 (94.5)	222 (91.4)	429 (92.5)
Male	10 (6.0)	3 (5.5)	20 (8.2)	33 (7.1)
Other	1 (0.6)	0	1 (0.4)	2 (0.4)
Age, *n* (%)				
< 30	0	4 (7.3)	28 (11.5)	32 (6.9)
30–39	21 (12.7)	11 (20.0)	85 (35.0)	117 (25.2)
40–49	54 (32.5)	13 (23.6)	51 (21.0)	118 (25.4)
50–59	57 (34.3)	24 (43.6)	55 (22.6)	136 (29.3)
≥ 60	34 (20.5)	3 (5.5)	24 (9.9)	61 (13.1)
Years in profession, *n* (%)				
< 2	19 (11.4)	17 (30.9)	48 (19.8)	84 (18.1)
2–4	29 (17.5)	9 (16.4)	59 (24.3)	97 (20.9)
5–9	41 (24.7)	11 (20.0)	38 (15.6)	90 (19.4)
≥ 10	77 (46.4)	18 (32.7)	98 (40.3)	193 (41.6)
Oral health education^a^, *n* (%)				
During professional education	87 (52.4)	33 (80.0)	63 (67.1)	294 (63.4)
At workplace	100 (60.2)	29 (52.7)	83 (34.2)	212 (45.7)
None	35 (21.1)	5 (9.1)	55 (22.6)	95 (20.5)

^a^Respondents could answer ‘Yes’ to both items, resulting in a cumulative count exceeding the total number of participants. The category ‘None’ denotes respondents who answered ‘No’ to both items

Table 2 presents frequencies and percentages of the background factors used for group comparisons in the statistical analyses, including professional role, respondents’ education in oral health, type of nursing home, use of Senior Alert and the ROAG-J, and how often the nursing staff are offered oral care training.

**Table 2:** Background factors used for statistical group comparisons

	*N*	%
Professional roles		
Managers	166	35.8
Coordinators	55	11.9
Nurses	243	52.4
Respondents’ oral health education**:**		
*During professional education*		
Yes	294	63.4
No	170	36.6
*At workplace*		
Yes	212	45.7
No	252	54.3
The nursing home’s use of:		
*Senior Alert*		
Yes	400	86.2
No	46	9.9
Don’t know	18	3.9
*ROAG-J*		
Yes	285	61.4
No	65	14.0
Don’t know	114	24.6
Type of nursing home		
Dementia care facility/ward	229	49.4
General nursing home	235	50.6
Oral care training for nursing staff		
Regular	114	24.6
Irregular	162	34.9
Don’t know	188	40.5

### Oral care training for nursing staff

A large proportion of respondents (40%) answered ‘Don’t know’ regarding how often nursing staff at their workplace are offered oral care training, with uncertainty being particularly high among nurses (67%) compared to coordinators (27%) and managers (6%). When excluding those who selected ‘Don’t know’, a total of 41% of respondents reported that nursing staff are offered regular training, either annually (24%) or every other year (17%). Significantly more managers (59%) than coordinators (25%) and nurses (15%) stated that nursing staff at their workplace received regular training (both < 0.001). Respondents who themselves had received oral health education at the workplace reported that their nursing staff received more regular training than those who had not (78% vs. 22%, *p* < 0.001).

### Residents’ oral health and needs of assistance with oral care

Overall, 46% of respondents perceived residents’ oral health as poor (fairly poor or very poor) and 83% estimated that ≥ 75% of residents required assistance with oral care. The respondents’ perceptions of nursing home residents’ oral health and their estimated need for assistance with daily oral care were analysed in relation to the background factors presented in Table 3.

**Table 3:** Perceptions of residents’ oral health and assistance needs, with group comparisons by background factors

	Oral health of the residents^a^			Residents in need of assistance with oral care^b^				
	*N*	Fairly /very poor (%)	*p*	*N*	About 25% or less (%)	About 50% (%)	About 75% or more (%)	*p*
Total	450	45.6		459	3.8	13.1	83.2	
Professional roles			**0.009** ^**c, e**^					0.084^c^
Nurses	240	51.2		241	3.3	12.9	83.8	
Managers	158	39.2		165	1.8	13.3	84.8	
Coordinators	52	38.5		53	11.3	13.2	75.5	
Respondents with oral health education:								
*During professional education*			0.218^d^					0.778^d^
Yes	287	43.6		292	2.7	12.3	84.9	
No	163	49.1		167	5.4	14.4	80.2	
*At workplace*			**0.046** ^d^					0.759^d^
Yes	205	41.5		211	3.8	14.2	82.0	
No	245	49.0		248	3.6	12.1	84.3	
Nursing home use of:								
*Senior Alert*			0.786^d^					0.509^d^
Yes	389	46.0		396	3.8	11.4	84.9	
No	45	42.2		46	0	15.2	84.8	
*ROAG-J*			0.604^d^					0.814^e^
Yes	279	45.5		283	3.9	11.0	85.2	
No	63	47.6		64	4.7	17.2	78.1	
Type of nursing home			0.640^d^					**< 0.001** ^d^
Dementia care facility/ward	221	44.8		229	2.2	10.5	87.3	
General nursing home	229	46.3		230	5.2	15.7	79.1	
Oral care training for nursing staff			**0.041** ^d^					0.209^d^
Regular	112	37.5		112	1.8	16.1	82.1	
Irregular	154	48.1		162	4.3	8.0	87.7	

Statistically significant values (*p* < 0.05) are reported in bold

^a^Response options: ‘Very poor’; ‘Fairly poor’; ‘Quite good’; ‘Very good’; ‘Don’t know’. The response ‘Don’t know’ was excluded from the analysis

^b^Response options: ‘None or very few’ and ‘About 25%’ (= About 25% or less); ‘About 50%’; ‘About 75%’ and ‘All or almost all’ (= About 75% or more); ‘No opinion’. The response ‘No opinion’ was excluded from the analysis

^c^ Kruskal–Wallis test, followed by pairwise comparisons when *p* < 0.05

^d^ Mann–Whitney U test

^e^Mann–Whitney U test: nurses >managers, *p* = 0.005; nurses vs. coordinators, *p* = 0.054; managers vs. coordinators, *p* = 0.898

### Oral health routines and practices in nursing homes

Respondents reported the following oral health routines at their nursing home, excluding those who answered ‘Don’t know’: use of the ROAG-J (81%), presence of oral health representatives (22%), and use of signing lists for oral care actions (32%). The latter refers to checklists where nursing staff can document whether daily oral care has been provided.

Several respondents were unsure whether oral health routines were implemented or present in their nursing home, answering ‘Don’t know’ regarding use of the ROAG-J (*n* = 114; 25%), signing lists (*n* = 50; 11%) and oral health representatives (*n* = 141; 30%). A substantial proportion of managers (36%) and coordinators (40%) did not know about the use of ROAG-J, compared to 13% of nurses. Additionally, 54% of nurses did not know whether their nursing home had oral health representatives, compared to 4% of managers and 7% of coordinators. Among respondents who reported having *oral health representatives* in their nursing home (*n* = 72), 76% stated that the representatives’ responsibilities were clearly or partially defined, and 72% reported that the representatives had received specific training for this role. Oral health routines in relation to background factors are presented in Table 4.

**Table 4:** Oral health routines in nursing homes, with group comparisons by background factors

	Use the ROAG-J			Use signing list for oral care actions			Presence of oral health representatives		
	*N*	Yes (%)	*p*	*N*	Yes (%)	*p*	*N*	Yes (%)	*p*
Total	350	81.4		414	31.6		323	22.3	
Professional role			**0.004** ^a, c^			0.144^a^			**0.006** ^a, d^
Nurses	211	84.4		218	28.9		112	32.1	
Managers	106	71.7		145	31.7		160	15.6	
Coordinators	33	93.9		51	43.1		51	21.6	
Respondents with oral health education:									
*During professional education*			0.191^b^			0.124^b^			0.786^b^
Yes	231	83.5		265	34.3		197	22.8	
No	119	77.3		149	26.8		126	21.4	
*At workplace*			0.407^b^			0.320^b^			0.495^b^
Yes	167	83.2		195	29.2		168	23.8	
No	183	79.8		219	33.8		155	20.6	
Nursing home use of:									
*Senior Alert*			**0.003** ^b^			**0.013** ^b^			0.825^b^
Yes	305	83.6		359	33.4		282	22.0	
No	36	61.1		41	14.6		33	24.2	
*ROAG-J*			-			**0.020** ^b^			**0.046** ^b^
Yes	-	-		265	34.3		195	26.2	
No	-	-		60	18.3		44	11.4	
Type of nursing home			0.132^b^			0.673^b^			**0.045** ^b^
Dementia care facility/ward	186	84.4		206	30.6		162	27.2	
General nursing home	164	78.0		208	32.7		161	17.4	
Oral care training for nursing staff			0.865^b^			**0.044** ^b^			1.000^b^
Regular	87	79.3		104	42.3		110	16.4	
Irregular	118	78.0		149	29.5		134	17.2	

Responses were: ‘Yes’, ‘No’, ‘Don’t know’

Descriptive data are presented as the number of respondents in each group and the percentage who answered ‘Yes’, excluding ‘Don’t know’ responses

Statistically significant values (*p* < 0.05) are reported in bold

^a^ Pearson Chi–Square test; if more than two groups, followed by pairwise comparisons when *p* < 0.05

^b^ Fisher’s exact test (two-sided)

^c^ Fisher’s exact tests: nurses > managers, *p* = 0.011; coordinators > managers, *p* = 0.008; nurses vs. coordinators, *p* = 0.186

^d^ Fisher’s exact tests: nurses > managers, *p* = 0.002; nurses vs. coordinators, *p* = 0.195; managers vs. coordinators, *p* = 0.392

*The dental care subsidy for frail older adults* was well-known, with 80% of respondents being very familiar and 12% somewhat familiar with it. Managers were significantly more aware of the subsidy (99%) compared to nurses (88%) and coordinators (90%) (both *p* < 0.001). Respondents who had received oral health education at the workplace had greater awareness of the subsidy than those who had not (99% vs. 86%, *p* < 0.001).

The following oral health practices were routinely (often/always) carried out in connection with nursing home admissions, excluding ‘Don’t know’ responses: asking residents about oral health problems (66%) and their contact with dental care services (80%), offering the dental care subsidy (92%) and documenting oral health and oral care in care plans (70%).

A substantial proportion of respondents (*n* = 109; 23%) did not know if residents’ oral health and oral care was documented in care plans. This lack of awareness was particularly high among nurses (41%) compared to coordinators (11%) and managers (2%). Table 5 presents the various oral health practices in relation to the background factors.

**Table 5:** Oral health practices in nursing homes, with group comparisons by background factors

	Asking about oral health problems at admission to nursing home			Asking about the contact with dental care services at admission to nursing home			Offering subsidy dental care support at admission to nursing home			Documenting oral health in care plans		
	*N*	Always/Often (%)	*p*	*N*	Always /Often (%)	*p*	*N*	Always/Often (%)	*p*	*N*	Always/Often (%)	*p*
Total	421	66.0		434	79.7		404	92.3		355	70.1	
Professional role			**< 0.001** ^a, c^			**< 0.001** ^a, d^			0.081^a^			0.361^a^
Nurses	228	56.6		227	70.0		198	90.4		144	68.8	
Managers	151	76.2		157	90.4		162	93.8		162	74.7	
Coordinators	42	81.0		50	90.0		44	95.5		49	59.2	
Respondents with oral health education:												
*During professional education*			0.058^b^			0.819^b^			0.186^b^			0.050^b^
Yes	268	70.1		276	80.4		256	93.4		227	72.2	
No	153	58.8		158	78.5		148	90.5		128	66.4	
*At workplace*			**< 0.001** ^b^			**< 0.001** ^b^			0.098^b^			**0.019** ^b^
Yes	196	71.9		203	85.2		204	92.6		175	74.9	
No	225	60.9		231	74.9		200	92.0		180	65.6	
Nursing home use of:												
*Senior Alert*			0.867^b^			0.711^b^			0.421^b^			0.186^b^
Yes	365	66.0		375	80.5		375	80.5		375	80.5	
No	39	61.5		44	77.3		44	77.3		44	77.3	
*ROAG-J*			0.129^b^			0.214^b^			**0.011** ^b^			0.123^b^
Yes	266	69.5		273	80.6		256	94.5		218	74.3	
No	59	61.0		59	78.0		56	91.1		48	68.8	
Type of nursing home			0.625^b^			**0.012** ^b^			**< 0.001** ^b^			0.112^b^
Dementia care facility/ward	212	65.1		218	84.9		207	94.7		178	72.5	
General nursing home	209	67.0		216	74.5		197	89.8		177	67.8	
Oral care training for nursing staff			**< 0.001** ^b^			**< 0.001** ^b^			0.110^b^			**0.018** ^b^
Regular	105	81.9		108	92.6		112	94.6		112	75.9	
Irregular	148	60.1		155	81.3		143	93.0		138	65.9	

Responses were: ‘Always’, ‘Often’, ‘Sometimes’, ‘Rarely’, ‘Never’, ‘Don’t know’

Descriptive data are presented as the number of respondents in each group and the percentage who answered ‘Always’ or ‘Often’, excluding ‘Don’t know’ responses

Statistically significant values (*p* < 0.05) are reported in bold

^a^ Kruskal–Wallis test; if more than two groups, followed by pairwise comparisons when *p* < 0.05

^b^ Mann–Whitney U test

^c^ Mann–Whitney U tests: nurses < managers, *p* < 0.001; nurses < coordinators, *p* = 0.004; managers vs. coordinators, *p* = 0.840

^d^ Mann–Whitney U tests: nurses < managers, *p* < 0.001; nurses < coordinators, *p* = 0.010; managers vs. coordinators, *p* = 0.315

### Collaboration with dental care services

The majority of the respondents (92%) reported that collaboration with dental care services worked well, rating it as very or quite good. Most respondents (93%) reported that their workplace offered dental care at the nursing home, with the majority (90%) stating that residents received mobile dental care in their apartments within the nursing home and 6% reporting that dental care was provided in a separate dental room at the facility. Multiple response options were possible for this question. One significant difference was found, with respondents working in dementia care facilities/wards reporting higher access to dental care compared to those in general nursing homes (97% vs. 92%, *p* = 0.038).

The majority of respondents knew whom to contact for dental care or for advice and help when older adults had oral health problems (always/often: 92%). This was more often reported by respondents who had received oral health education at the workplace than by those who had not (always/often: 96% vs. 89%, *p* = 0.003), and more often in nursing homes where staff had received regular oral care training compared to those with irregular training (always/often: 95% vs. 90%, *p* = 0.003).

### Open-ended question about collaboration between nursing care and dental care services

A total of 99 respondents (21%) provided input on the open-ended question regarding their views on an ideal structure for collaboration between nursing care and dental care services, including 61 nurses (25%), 31 managers (19%) and 7 coordinators (13%).

Overall, about half of the respondents (*n* = 52; 53%) viewed the current collaboration with dental care services in their workplace positively, noting that most residents benefited from home dental care due to difficulties traveling to dental clinics. Both public and private dental care providers were reported to offer services within the nursing homes. However, 12 respondents (12%) found this structure confusing and overlapping, as they were uncertain who was responsible for dental care and whom to contact regarding residents’ oral health needs. In addition, 22 nurses (36%) also expressed a desire for improved communication with dental professionals, including clearer documentation and follow-up plans. Furthermore, 15 respondents (15%) emphasized the need for more continuous and mandatory oral health training for nursing staff, along with increased support from dental professionals in daily nursing care.

### Barriers and facilitators to oral care in nursing homes

Table 6 presents the extent to which respondents experienced various barriers to oral care.

**Table 6:** Perceived extent of barriers to oral care in nursing homes

Barriers	*N*	Disagree^a^ (%)	Partly agree^b^ (%)	Completely agree^c^ (%)
Oral care is time-consuming	447	39.4	37.4	23.3
Nursing staff perceive oral care as practically difficult to perform	448	28.1	41.7	30.1
Nursing staff perceive oral care as a personal intrusion	436	48.2	31.0	20.9
The residents resist assistance with oral care	442	28.3	43.7	28.1
Oral health routines are lacking or are unclear	447	65.8	19.0	15.2
Oral care products are missing (toothbrush, toothpaste etc.)	448	91.5	7.6	0.9
Nursing staff lack knowledge and training in oral care	448	43.1	35.7	21.2

The response ‘No opinion’ was excluded in the analysis

^a^ Disagree: responses ‘Not at all’ and ‘A little’

^b^ Partly agree: response ‘Partly’

^c^ Completely agree: responses ‘Quite a lot’ and ‘Completely’

The most commonly perceived barriers were oral care being practically difficult for nursing staff to perform and residents resisting assistance with oral care (partly/completely agree: 72%, for both).

Nurses and managers agreed to a higher extent (partly/completely) that *oral care is time-consuming* compared to coordinators (64% and 60% vs. 48%, nurses > coordinators: *p* = 0.007). *Lacking or unclear oral health routines* were perceived as a barrier to a greater extent by nurses than by managers and coordinators (partly/completely agree: 45% vs. 21% and 25%, both *p* < 0.001). This barrier was less frequently perceived by respondents in dementia care facilities/wards compared to general nursing homes (disagree: 72% vs. 59%, *p* = 0.023) and by respondents in nursing homes using Senior Alert than those that do not use the register (disagree: 68% vs. 49%, *p* = 0.002). The barrier of *nursing staff lacking knowledge and training in oral care* was perceived to a higher extent by nurses compared to managers and coordinators (partly/completely agree: 69% vs. 43% and 46%, both *p* < 0.001).

###  Open-ended question about barriers and facilitators to oral care

A total of 132 respondents (28%) provided input to the open-ended question regarding barriers and facilitators in providing oral care in nursing homes, including 69 nurses (28%), 47 managers (28%) and 16 coordinators (29%).

Content analysis revealed that among the 103 responses about *barriers*, the most commonly mentioned issue was residents resisting assistance with oral care (*n* = 57; 55%). This care-resistant behaviour, such as not being willing to open one’s mouth, was often attributed to cognitive impairment, which made it difficult for residents to understand instructions and stay motivated (*n* = 38; 67%). Other contributing factors to resistance included the need for privacy (*n* = 7; 12%), as some residents preferred to brush their teeth independently, as well as pain or discomfort during oral care (*n* = 3; 5%) and language barriers (*n* = 5; 9%). Respondents also reported that nursing staff sometimes perceived oral care as a personal intrusion (*n* = 15; 15%) and lacked knowledge and training in this area (*n* = 18; 17%). Furthermore, 18 nurses (33%) reported that lack of time and personnel was a major challenge, whereas this concern was rarely mentioned by managers and coordinators (*n* = 2; 4%).

Among the 75 responses that addressed *facilitators*, the most commonly reported factor was the need for more regular training and guidance in oral care for nursing staff (*n* = 41; 55%). Training was particularly emphasized for new staff, temporary workers and relatives, focusing on both practical tips and strategies for encouraging people with dementia to participate in oral care. Another facilitator mentioned was the need for active and effective collaboration with dental care services (*n* = 21; 28%), including dental care professionals visiting nursing homes to provide dental care and educate nursing staff. Some respondents (*n* = 13; 17%) also stressed the importance of having clear and well-followed oral health routines and guidelines as a facilitator.

### Oral health in the quality register Senior Alert

The majority of respondents rated working with the quality register Senior Alert as functioning well, with 47% stating ‘quite well’ and 33% ‘very well’. A total of 380 respondents (82%) were familiar with performing risk assessments in Senior Alert (Table 7). Among the five risk assessments in Senior Alert, the oral health assessment ROAG-J was considered the most difficult to perform. Coordinators found it significantly less challenging than managers and nurses (both *p* < 0.001) (Table 7).

**Table 7:** The experience of working with the quality register Senior Alert (SA)

	Managers^a^ *n* (%)	Coordinators^a^ *n* (%)	Nurses^a^ *n* (%)	Total^a^ *n* (%)
Experience in performing risk-assessment in SA	119 (71.7)	38 (69.1)	223 (91.8)	380 (81.9)
Participate in SA team meetings	115 (87.1)	27 (62.8)	172 (97.2)	314 (89.2)
Difficulty^b^ assessing risks for:				
Pressure ulcers	1 (0.9)	0	6 (2.7)	7 (1.9)
Malnutrition	2 (1.8)	0	9 (4.1)	11 (3.0)
Falls	1 (0.9)	0	7 (3.2)	8 (2.2)
Oral health	30 (27.5)	6 (16.2)	63 (28.6)	99 (27.0)
Bladder dysfunction	13 (16.5)	2 (7.7)	48 (29.4)	63 (23.5)
Discuss^c^ at meetings residents’ risk for:				
Pressure ulcers	104 (92.9)	26 (100)	153 (90.0)	283 (91.9)
Malnutrition	106 (94.6)	26 (100)	157 (92.4)	289 (93.8)
Falls	107 (95.5)	26 (100)	161 (94.7)	294 (95.5)
Oral health	91 (81.3)	24 (96.0)	120 (71.0)	235 (76.8)
Bladder dysfunction	67 (64.4)	14 (66.7)	56 (36.4)	137 (49.1)

^a^ Respondents who selected the response ‘No opinion’ were not included in the table

^b^ Response options: ‘Quite difficult’ and ‘Very difficult’

^c^ Response options: ‘Always’ and ‘Often’

A total of 353 respondents (76%) reported that their nursing home held team meetings to discuss the risks of frail older adults, with the majority (89%) attending the meetings themselves. The most commonly discussed risks in Senior Alert included falls, malnutrition and pressure ulcers, while oral health risks and bladder dysfunction were addressed the least frequently (Table 7).

### Open-ended question regarding strengths and challenges of Senior Alert

A total of 120 respondents (26%) responded to the open-ended question regarding additional strengths and challenges of the quality register Senior Alert, including 63 nurses (26%), 50 managers (30%) and 7 coordinators (13%).

Of these, 31 respondents (26%) only stated that they no longer actively used Senior Alert in their nursing home, and they were therefore excluded from further content analysis. Among the remaining responses, approximately half (*n* = 46; 52%) viewed Senior Alert positively, describing it as an effective preventive and systematic approach that offers a comprehensive understanding of individuals’ care needs and serves as a reliable tool for ensuring high-quality care and support. However, challenges were also noted, with 25 respondents (28%) describing the system as time-consuming and requiring extensive documentation, often leading to duplicate records in different digital systems. In addition, 14 respondents (16%) questioned the purpose and usefulness of Senior Alert, noting that the clinical picture of individuals often did not align with the risks identified through the assessments.

### The ROAG-J: experience and training

A total of 257 respondents (55%) reported having experience in performing the ROAG-J. There was a significant difference between professional groups: 81% of nurses had experience with the ROAG-J, compared to 20% of managers and 45% of coordinators (both *p* < 0.001). Additionally, among those with experience of performing the ROAG-J, 60% had received education in its use, with 27% having been trained by dental professionals.

**Fig. 1:**
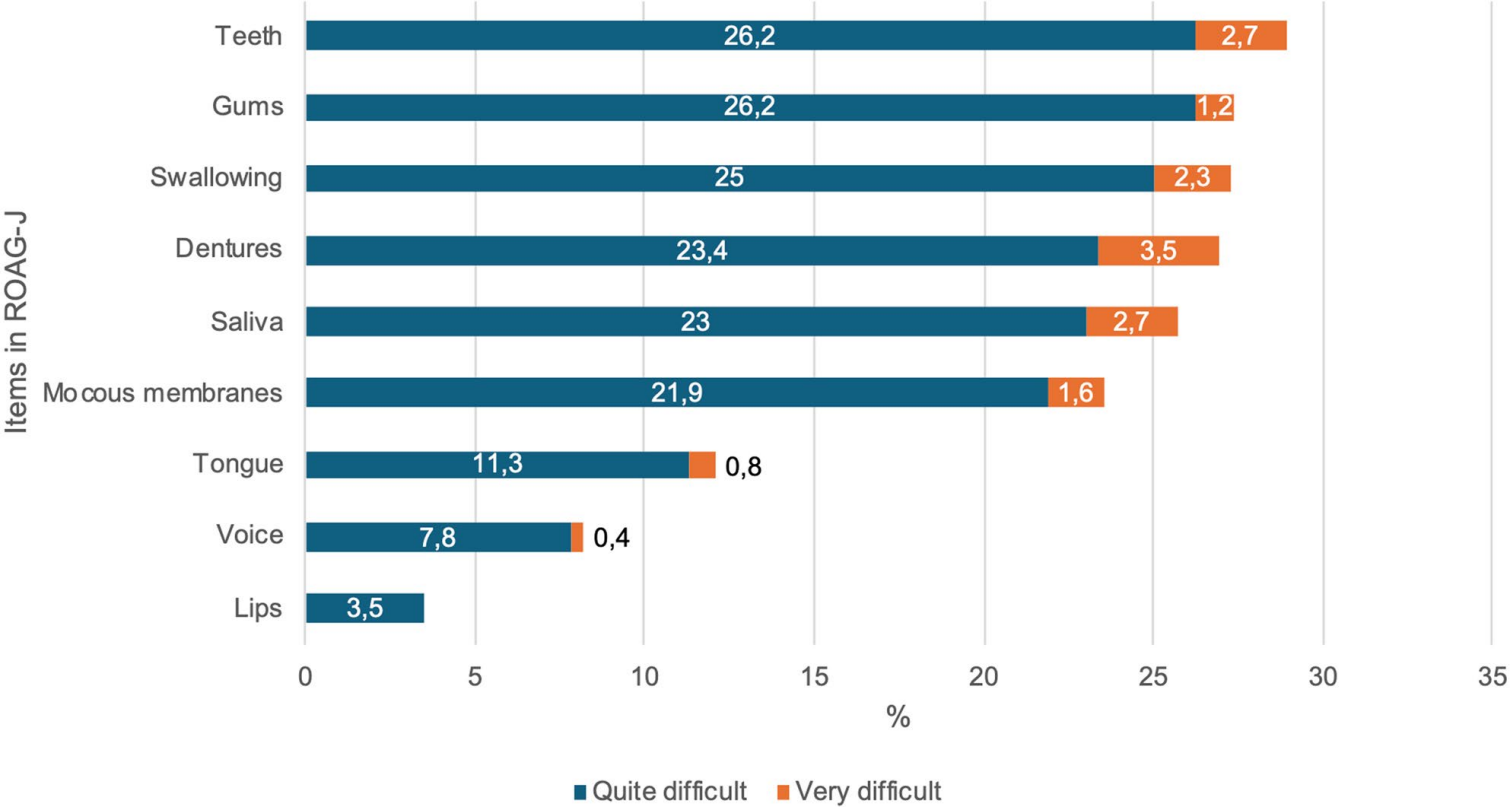
Percentage of respondents finding each ROAG-J item difficult to assess. Total responses: *n* = 256 (*n* = 255 for lips)

Figure 1 presents the percentage of respondents with experience of performing the ROAG-J who found each item quite or very difficult to assess, excluding “don’t know” responses.

The most challenging item to assess was *teeth* (29%), followed by *gums* and *swallowing* (both 27%). The least difficult items to assess were *lips* followed by *voice* (Fig. 1).

Of the respondents who reported that the ROAG-J was used in their nursing homes (*n* = 258), 73% thought that it worked well for nursing staff to conduct the assessments. A total of 88 respondents (19%) did not know whether nursing staff in their nursing homes had received training in the ROAG-J. Among those who did know (*n* = 197), 56% reported that 25% or less of the staff had been trained in its use. Regarding the availability of necessary instruments to perform the ROAG-J, such as a flashlight and mouth mirror, 56% confirmed that these were available. ROAG-J assessments were primarily performed by nurse assistants (*n* = 191; 67%), followed by registered nurses (*n* = 162; 57%), with respondents being able to select multiple professions for this question. When asked which profession was most suitable for performing the ROAG-J, most respondents selected nurse assistants (39%), followed by registered nurses (32%).

### Open-ended question regarding the strengths and challenges of the ROAG-J

A total of 66 respondents (14%) answered the open-ended question regarding the advantages and disadvantages of the ROAG-J, including 45 nurses (18%), 18 managers (11%) and 3 coordinators (5%).

Among the nurses, 15 (33%) stated that nurse assistants often lacked the routine and necessary training to perform the ROAG-J, which could increase the risk of incorrect or missed assessments. Furthermore, 17 nurses (38%) reported that they carried out the ROAG-J themselves instead of delegating the task to nurse assistants. In addition, 21 respondents (32%) expressed that performing the assessments was challenging, particularly on uncooperative residents such as individuals with dementia.

## Discussion

The main findings of this study stress the urgent need to prioritize the oral health of frail older adults in nursing homes. Nearly half of the respondents perceived residents’ oral health as poor, and just over 80% estimated that the vast majority (about 75% or more) of residents required assistance from caregivers with daily oral care. A previous Swedish study also examined these aspects of oral care. Of over 20,000 nursing home residents assessed by dental hygienists, 78% were found to have poor oral hygiene and to require assistance with daily oral care. Nevertheless, only 7% were reported to have received such support [37].

The present study revealed a difference between the professional leadership roles, with nurses perceiving residents’ oral health as poorer than managers and coordinators. Given that nurses work closer to residents, the discrepancy may suggest that managers and coordinators underestimate the residents’ oral health problems and their need for oral care and dental treatment, a finding that also been reported in other studies [38, 39]. Research also indicates that registered nurses in Swedish nursing homes do not have a clear responsibility for the residents’ oral health, as the daily oral care is primarily handled by nurse assistants [21, 22]. Since nurse assistants are supervised by managers and coordinators, the level of managerial engagement may influence the extent to which oral health is prioritized among the nursing tasks. It has also been reported that managers rarely take explicit responsibility for residents’ oral care and lack insight into the daily oral care tasks performed by nurse assistants [19]. Therefore, it is crucial to ensure that management is well informed about residents’ oral health status.

The present study revealed that the greatest perceived challenges regarding oral care in nursing homes were residents’ unwillingness to cooperate and the difficulty of performing this task. Several other studies have similarly identified residents’ poor cooperation as the main barrier, which can contribute to neglecting oral care [21, 40, 41]. In fact, failure to provide oral care has been shown to be the nursing care task most commonly neglected by nursing staff [42]. Moreover, research has demonstrated that residents who require assistance with daily oral care and those who resist care have significantly poorer oral hygiene than independent residents [43, 44]. Assisting residents with natural teeth has been shown to be time-consuming and challenging, especially regarding residents with cognitive and physical impairments [20]. This aligns with the present study, where the respondents also estimated that there was a greater need for assistance with oral care among residents in dementia care facilities/wards compared to residents in general nursing homes. A close relationship combined with a person-centred approach is crucial to overcome residents’ reluctance towards oral care, particularly among individuals with dementia [40]. However, nursing staff often lack the training required to meet this challenge [45].

In this study, more than half of the respondents perceived the lack of oral care training among nursing staff as a barrier, and more frequent staff training was viewed as an important facilitator. In contrast, only 41% estimated that nursing staff had received such training. This finding aligns with previous research showing that less than half of caregivers had received oral care education [41, 46]. Moreover, the gap between the need for training and its actual provision is a significant organizational barrier [41]. Educational interventions, including hands-on training for caregivers, could be effective in improving older adults’ oral health [47]. In the present study, respondents from nursing homes where nursing staff received regular oral care training perceived oral care routines as more established and residents’ oral health as better compared to settings where training was offered irregularly. This suggests that regular training in oral care can positively influence residents’ oral health. The findings also indicate the importance of workplace oral health education for management staff, as this was associated with a more frequent use of various oral health practices. In contrast, no significant differences were observed among respondents who had received oral health education during their professional education, indicating that workplace training may have a greater impact. Altogether, these results emphasize the importance of continuous training among nursing home staff to maintain up-to-date oral health knowledge and practices.

In this study more nurses than managers and coordinators perceived that oral health routines in nursing homes were inadequate or unclear, and that nursing staff were lacking knowledge and training in oral care. Interestingly, in the open-ended responses several registered nurses also mentioned time and staffing shortages as obstacles to oral care, challenges that were rarely cited by managers and coordinators. Our previous qualitative study similarly found that nursing staff felt managers did not prioritize oral health and did not allocate sufficient time and resources for staff training [21]. This indicates that managers and coordinators may not fully recognize the barriers and needs related to oral care and may not prioritize this task to the same extent as nurses. The results also indicate a possible lack of communication between professions and lack of structure regarding oral health practices, as more nurses than managers and coordinators answered ”Don’t know” to whether nursing staff had received training or whether the nursing home had oral health representatives. This likely reflects that nurses do not organize or provide training for nurse assistants, whereas managers, who had the fewest “Don’t know” responses, typically do. Furthermore, more managers and coordinators than nurses answered ”Don’t know” about whether the ROAG-J was being used, suggesting they may be less involved in oral health assessments than nurses.

Oral health routines that could be emphasized further in nursing homes include the use of a signing list which was reported by approximately one-third of the respondents in this study. This is important because research has shown that preventive oral care actions, such as assistance with toothbrushing, are often insufficiently provided despite the detection of oral health problems [37, 48]. A signing list can also help identify residents who resist oral care, enabling nursing staff to seek advice from dental care services in such cases. In addition, the role of oral health representatives, often held by nurse assistants, was by the respondents reported to be used to a limited extent (22%). These representatives are intended to have enhanced competence in oral health and support other nursing staff through guidance and education. This role is particularly important given the high staff turnover and the presence of many temporary workers. Additionally, research has shown that having oral health representatives increases the number of residents receiving assistance with daily oral care [49].

The present study also identified inadequate communication and documentation regarding residents’ oral health and follow-up recommendations from dental professionals to nurses, a challenge that also has been emphasized in previous research [50]. In 2019, the Swedish National Board of Health and Welfare reported that poor integration between dental care and healthcare – partly due to separate laws and documentation systems – hinders coordination and the provision of holistic care for older adults in Sweden [23]. To address these challenges, improved information exchange, such as through collaborative forums for mutual learning, could promote more integrated care [23, 51].

A positive finding from this study was that the vast majority (over 90%) of respondents reported good availability of dental care in the nursing homes and knew whom to contact within dental care services in case of problems or questions. Additionally, 80% stated that residents were asked about their dental care contacts upon admission to the nursing home, which is particularly important given the clear decline in dental care utilization among individuals with cognitive impairments [8]. Another encouraging result in this study was that the dental care subsidy for older adults in Swedish nursing homes was well known among respondents, with 92% reporting that it was offered at admission. This subsidy helps ensure that older adults can afford dental treatment, reducing the risk of them neglecting oral health and developing oral diseases.

Working with the quality register Senior Alert facilitates the structured collection of care information and systematic follow-up to ensure high-quality care [52]. The present study showed that the preventive care process regarding oral health in Senior Alert was well integrated and was generally perceived to function effectively. Using Senior Alert also appeared to support greater inclusion of oral health in care plans and a more frequent use of signing lists for oral care actions. Furthermore, Senior Alert promoted increased communication about oral health, as it was regularly discussed at team meetings where management also actively participated. Such involvement may strengthen their awareness of the importance of residents’ oral health. However, some challenges were also identified. Working with Senior Alert was perceived as time-consuming, particularly its documentation, requiring managers to allocate sufficient time for staff. In addition, oral care training for staff was often reported as irregular or insufficient. This suggests that while Senior Alert is broadly implemented and generally well received, practical barriers may limit its potential impact.

This study also showed that the ROAG-J was frequently used by nursing staff, which could indicate regular monitoring, preventive care and timely contact with dental care when properly implemented. This is essential for the early detection of problems and for improving the quality of care in nursing homes, as serious oral health issues can otherwise develop and negatively affect the overall health and quality of life of residents. However, challenges remain, as almost half of the respondents reported that the essential equipment, flashlight and mouth mirror, were missing, and over half estimated that only a small proportion of nursing staff had received training in using the ROAG-J. Expanding training and ensuring access to necessary tools are crucial to prevent missed or incorrect assessments, especially since the ROAG-J also was considered the most challenging of all risk assessments in Senior Alert.

As the ROAG-J is an objective assessment tool designed to facilitate the detection of oral health problems, self-perceived issues reported by older adults, such as dental pain, are not captured. Notably, many respondents (66%) stated that residents were asked about perceived oral health problems upon admission to the nursing home. This practice should be considered before each ROAG-J assessment, both to increase the involvement of the older adult and to ensure a more comprehensive evaluation of their oral health needs. Since residents’ oral health may deteriorate over time, ROAG-J assessments should be performed upon admission and at least every six months to ensure timely detection of changes and appropriate care.

### Strengths and limitations

The response rate in the present study was low (32%), which may have introduced bias and potentially influenced the results. However, it is higher than in previous studies conducted in the same field [39, 53]. Online surveys generally yield lower response rates than other survey methods but are often more efficient and practical for reaching large samples [54]. Moreover, a large-scale evaluation has shown that striving for a high response rate provides little or no reduction in non-response bias [55]. However, in the present study, due to the anonymous nature of the questionnaire, no comparison between respondents and non-respondents could be made, which limits the ability to assess potential non-response bias. Therefore, the results should be interpreted with caution and considered indicative rather than conclusive.

A strength of this study is that the questionnaire was distributed to a large number of individuals across a geographically wide area, encompassing numerous nursing homes. This resulted in a substantial number of completed responses, which may enhance the generalizability of the findings. The response rate was also consistent across all professional roles. Notably, the low response rate may itself also reflect the limited attention that oral health receives within the nursing care of frail older adults. It is also likely that the respondents in this study are those who are more positive and interested in oral health, which could introduce potential bias. A further limitation is that nursing home leaders’ perceptions of residents’ oral health may be based on second-hand information from nursing staff, which could affect validity. Nevertheless, these perceptions are important as they influence care priorities and the establishment of routines.

Additionally, 12% of the respondents were excluded because they worked in home care or home healthcare rather than nursing homes. Since the email invitation to the online survey clearly stated that participants should be employed in nursing homes, it is likely that many individuals who did not meet the inclusion criteria chose not to proceed with the survey. Therefore, the actual response rate among the intended target group may have been higher than 32%, potentially strengthening the representativeness of the results.

To minimize non-response bias, three reminders were sent out, and respondents had the option to start, save and complete the survey at a later time. One limitation of the study is that the rather long questionnaire may have been challenging for some staff to complete, particularly given their demanding working conditions. A further limitation is that, because responses were given anonymously, it was not possible to determine how many individual nursing homes were represented. This limits the ability to draw conclusions about, for example, the share of nursing homes that are using a specific routine.

Given the descriptive and exploratory nature of this analysis, no correction for multiple testing was applied. As a result, there is a risk of false-positive findings, and the results warrant verification in future studies. Nevertheless, the study provides valuable insights, particularly as few studies have examined oral health management in nursing homes from an organizational perspective.

## Conclusion

The study stresses the importance of prioritizing oral health in the nursing care of frail older adults. Many residents were perceived to have poor oral health and to require assistance with daily oral care, with several complex barriers hindering oral care provision. To improve residents’ oral health, it is essential to establish clear routines, strengthen collaboration with dental care services, and ensure that nursing staff receive regular, mandatory oral care training. Management’s active involvement is crucial to support and sustain these efforts. Senior Alert’s structured approach seems to have the potential to enhance the engagement of nursing home professionals in managing residents’ oral health.

## Supplementary Information

Supplementary Material 1

Supplementary Material 2

## Data Availability

The datasets used and analysed during the present study are available from the corresponding author upon reasonable request. Corresponding author: Lisa Bellander, lisa.bellander@gu.se.
