# Supplementary material for: Oral health care for frail older adults in nursing homes from a management perspective: a survey-based study
Source: BMC Oral Health. 2025 Dec 14;25:1903. doi: 10.1186/s12903-025-07327-x (PMC12703933; doi:10.1186/s12903-025-07327-x)
Supplement: Supplementary file 2 — Supplementary Material 2 [file 12903_2025_7327_MOESM2_ESM.docx]

**Questionnaire:**

**Oral health and oral care in nursing care for frail older adults**

**Section A: Demographics**

1. **Where do you work?**
   - Halland
   - Västra Götaland
2. **Do you work in a nursing home?**

*Filter question: Respondents who answer “No” will be directed to the end of the survey. Only respondents who answer “Yes” will continue with the survey.*

- - Yes
  - No, only in short-term care
  - No, only in home care or home healthcare

1. **Do you primarily work in a dementia care facility/ward?**
   - Yes
   - No
2. **Ownership of the nursing home:**
   - Public
   - Private
3. **Gender:**
   - Female
   - Male
   - Other
4. **How old are you?**
   - 19 years or younger
   - 20–29 years
   - 30–39 years
   - 40–49 years
   - 50–59 years
   - 60 years or older
5. **What is your professional role?**
   - Registered nurse
   - Manager (unit manager or operations manager)
   - Coordinator/team leader
   - Other
6. **How long have you held this professional role in nursing care for older adults?**
   - Less than 1 year
   - 1 year
   - 2–4 years
   - 5–9 years
   - 10 years or more
7. **What is your vocational or professional education?**
   - Registered nurse
   - Nurse assistant
   - Social worker
   - Physiotherapist
   - Occupational therapist
   - Other
8. **Have you received oral health education as part of your professional education?**
   - Yes
   - No
9. **Have you received oral health education at your current workplace?**
   - Yes
   - No
10. **How often do you visit a dentist or dental hygienist?**
    - At least once a year
    - About every other year
    - Less often than every other year
    - Only for emergency visits
    - Not at all
11. **Are you afraid of (or uncomfortable with) visiting the dentist?**
    - No
    - Yes, a little
    - Yes, rather
    - Yes, very

**Section B: Oral health care needs, barriers and facilitators to oral care**

1. **How do you perceive nursing home residents’ oral health?**
   - Very good
   - Quite good
   - Fairly poor
   - Very poor
   - Don’t know
2. **How many nursing home residents do you estimate need assistance or help with daily oral care?**
   - None or very few
   - About 25%
   - About 50%
   - About 75%
   - All or almost all
   - No opinion
3. **To what extent do the following statements about barriers to oral care apply?**
   (Not at all, A little, Partly, Quite a lot, Completely, No opinion)

- Oral care is time-consuming
- Nursing staff perceive oral care as practically difficult to perform
- Nursing staff perceive oral care as a personal intrusion
- Nursing home residents resist assistance with oral care
- Oral health routines are lacking or are unclear
- Oral care products are missing (toothbrush, toothpaste etc.)
- Nursing staff lack knowledge and training in oral care

1. **If you wish, please elaborate: What barriers are there and what could help facilitate oral care in nursing homes?**
   *(Open-ended response)*

**Section C: Oral health routines, oral care training and cooperation with dental care services**

1. **Are the nursing home residents asked about oral health problems upon admission to the nursing home?**
   - Always
   - Often
   - Sometimes
   - Rarely
   - Never
   - Don’t know
2. **Are the nursing home residents asked about their contact with dental care services upon admission to the nursing home?**
   - Always
   - Often
   - Sometimes
   - Rarely
   - Never
   - Don’t know
3. **Is there a signing list for completed oral care actions for the nursing home residents who need assistance with oral care?**
   - Yes
   - No
   - Don’t know
4. **Is the nursing home residents’ oral health and oral care documented in their care plans?**
   - Always
   - Often
   - Sometimes
   - Rarely
   - Never
   - Don’t know
5. **Are you familiar with the dental care subsidy (Nödvändig tandvård) for frail older adults?**

*Filter question: Only respondents who answered “Yes” received question 6.*

- Yes, very familiar
- Yes, somewhat familiar
- No

1. **Is the dental care subsidy (Nödvändig tandvård) for frail older adults usually offered upon admission to the nursing home?**

- Always
- Often
- Sometimes
- Rarely
- Never
- Don’t know

1. **Are there oral health representatives in the nursing home?**

*Filter question: Only respondents who answered “Yes” received questions 8–9.*

- Yes
- No
- Don’t know

1. **Are the responsibilities of the oral health representatives clearly defined?**

- Yes
- Partially
- No
- Don’t know

1. **Have the oral health representatives received specific training related to their role?**

- Yes
- No
- Don’t know

1. **How often is regular nursing staff offered oral care training?**

- Every year
- Every other year
- Less frequently than every other year
- Never
- Don’t know

1. **Do you know who to contact within dental care services if a nursing home resident has oral health problems or if advice and help are needed?**

- Always
- Often
- Sometimes
- Rarely
- Never
- Don’t know

1. **If a nursing home resident has oral health problems, can they receive dental care from a dentist/dental hygienist at the nursing home?**

*(Multiple answers possible)*

- Yes, in the residents’ apartments
- Yes, in a separate treatment room in the nursing home
- Yes, in a mobile dental unit outside the nursing home
- No
- Don’t know

1. **How would you rate the collaboration with dental care services?**

- Very good
- Quite good
- Fairly poor
- Very poor
- Don’t know

1. **If you wish, please elaborate: In an ideal world, how should collaboration between nursing homes and dental care services be structured?**
   *(Open-ended response)*

**Section D: Senior Alert**

1. **Do you have experience of performing risk assessments in Senior Alert?**

*Filter question: Only respondents who answered “Yes” received question 2.*

- - Yes, I currently perform risk assessments.
  - Yes, I have performed risk assessments in the past.
  - No

1. **How do you perceive performing risk assessments in the following areas of Senior Alert?**
   (Very easy, Quite easy, Quite difficult, Very difficult, No opinion)
   - Pressure ulcers
   - Malnutrition
   - Falls
   - Oral health
   - Bladder dysfunction
2. **Does the nursing home where you currently work use Senior Alert?**

*Filter question: Respondents who answered “No” or “Don’t know” received questions 4–5 and then proceeded to section E. Respondents who answered “Yes” continued with question 5.*

- - Yes
  - No
  - Don’t know

1. **Would you like the nursing home to use Senior Alert?**
   - Yes
   - No
   - Don’t know
2. **If you wish, please describe what you think works well and what does not work so well regarding Senior Alert.**
   *(Open-ended response)*
3. **How well do you think working with Senior Alert works?**
   - Very well
   - Quite well
   - Fairly poorly
   - Very poorly
   - Don’t know
4. **Does the nursing home where you work hold team meetings where the risks identified in Senior Alert are discussed?**

*Filter question: Only respondents who answered “Yes” received questions 8–9.*

- - Yes, at least once a month
  - Yes, less often than once a month
  - No
  - Don’t know

1. **Do you participate in these team meetings yourself?**

*Filter question: Only respondents who answered “Yes” received question 9.*

- - Yes
  - No

1. **When residents’ risks are discussed in Senior Alert team meetings, how often are the following areas addressed?**
   (Always, Often, Sometimes, Rarely, Never, No opinion)
   - Pressure ulcers
   - Malnutrition
   - Falls
   - Oral health
   - Bladder dysfunction
2. **Does your nursing home use any alternative methods instead of team meetings to discuss residents’ risks in Senior Alert?**
   - Yes
   - No
   - Don’t know
3. **If another method is used, please describe it here:**
   *(Open-ended response)*

**Section E: ROAG-J**

1. **Do you have experience performing oral health assessments using the ROAG-J?**

*Filter question: Only respondents who answered “Yes” received questions 2–3.*

- - Yes
  - No

1. **How do you find assessing the following risk items in ROAG-J?**
   (Very easy, Quite easy, Quite difficult, Very difficult, No opinion)
   - Voice
   - Lips
   - Mucous membranes
   - Tongue
   - Gums
   - Teeth
   - Dentures
   - Saliva
   - Swallowing
2. **Have you received training in ROAG-J?**

- Yes, from dental care professionals
- Yes, through digital training
- No

1. **Are oral health assessments using ROAG-J conducted at the nursing home where you currently work?**

*Filter question: Only respondents who answered “Yes” received questions 5–10.*

- Yes
- No
- Don’t know

1. **How well do you think the nursing staff find that performing ROAG-J assessments works?**

- Very well
- Quite well
- Fairly poorly
- Very poorly
- Don’t know

1. **Please elaborate on what you think works well and not so well when performing ROAG-J assessments.***(Open-ended response)*
2. **Are instruments (flashlight and mouth mirror) available for ROAG-J assessments?**

- Yes
- No
- Don’t know

1. **Who most often performs ROAG-J assessments at the nursing home where you currently work?**

*(Multiple answers possible)*

- Registered nurses
- Nurse assistants/Care aides
- Oral health representatives
- Others
- Don’t know

1. **Which professional group do you think is most suitable for performing ROAG-J assessments?**

- Registered nurses
- Nurse assistants/Care aides
- Oral health representatives
- Others
- No opinion

1. **What proportion of the nursing staff do you estimate has received training in ROAG-J?**

- None or very few
- About 25%
- About 50%
- About 75%
- All or almost all
- No opinion
